# Defining usual care comparators when designing pragmatic trials of complex health interventions: a methodology review

**DOI:** 10.1186/s13063-024-07956-7

**Published:** 2024-02-12

**Authors:** Katrina M. Turner, Alyson Huntley, Tom Yardley, Sarah Dawson, Shoba Dawson

**Affiliations:** Population Health Sciences, Bristol Medical School, University of Bristol, Bristol, UK

**Keywords:** Methodology, Randomised controlled trials, Usual care, Comparator arms, Complex health interventions

## Abstract

**Background:**

Pragmatic trials evaluating complex health interventions often compare them to usual care. This comparator should resemble care as provided in everyday practice. However, usual care can differ for the same condition, between patients and practitioners, across clinical sites and over time. Heterogeneity within a usual care arm can raise methodological and ethical issues. To address these it may be necessary to standardise what usual care entails, although doing so may compromise a trial’s external validity. Currently, there is no guidance detailing how researchers should decide the content of their usual care comparators. We conducted a methodology review to summarise current thinking about what should inform this decision.

**Methods:**

MEDLINE, Embase, CINAHL and PsycINFO were searched from inception to January 2022. Articles and book chapters that discussed how to identify or develop usual care comparators were included. Experts in the field were also contacted. Reference lists and forward citation searches of included articles were screened. Data were analysed using a narrative synthesis approach.

**Results:**

One thousand nine hundred thirty records were identified, 1611 titles and abstracts screened, 112 full texts screened, and 16 articles included in the review. Results indicated that the content of a usual care comparator should be informed by the aims of the trial, existing care practices, clinical guidelines, and characteristics of the target population. Its content should also be driven by the trial’s requirements to protect participants, inform practice, and be methodologically robust, efficient, feasible and acceptable to stakeholders. When deciding the content of usual care, researchers will need to gather information about these drivers, balance tensions that might occur when responding to different trial objectives, and decide how usual care will be described and monitored in the trial.

**Discussion:**

When deciding the content of a usual care arm, researchers need to understand the context in which a trial will be implemented and what the trial needs to achieve to address its aim and remain ethical. This is a complex decision-making process and trade-offs might need to be made. It also requires research and engagement with stakeholders, and therefore time and funding during the trial’s design phase.

**Methodology review registration:**

PROSPERO CRD42022307324.

**Supplementary Information:**

The online version contains supplementary material available at 10.1186/s13063-024-07956-7.

## Background

Pragmatic trials evaluating the effectiveness of complex health interventions often evaluate new or modified treatments against a usual care comparator arm. As these trials aim to inform policy and practice in real-world settings, it is important that the usual care comparator resembles care normally provided in everyday practice [1]. Achieving this, however, might not be straightforward. Whilst the term usual care implies that there is consistent practice against which an intervention can be assessed, which may be the case where there is high-level evidence for a particular treatment [2], usual care can differ for the same condition, between patients and practitioners, across clinical sites, countries and over time [3].

Some researchers argue that to strengthen a trial’s external validity, the potential heterogeneity in usual care should be accepted and the trial’s usual care arm should include the full range of treatments available [4, 5]. The problem with this approach is that can make interpretation of trial findings difficult, with a potential lack of clarity about what the intervention is being compared against. Such detail is needed as the content and quality of the usual care arm will affect the effect size found, and therefore how effective the intervention is determined to be [6]. It is also problematic because, as Mant [7] comments, even if usual care is fully described, interpreting a trial’s results and applying them to clinical practice is very difficult if the care reported varies in content and quality.

The heterogeneity in usual care can also raise methodological and ethical problems. For example, trials often aim to detect clinically important effect sizes, which are based on the predicted difference between the effectiveness of the control and intervention groups. Not understanding what usual care consists of, therefore, undermines the basis on which sample sizes are calculated [8]. This lack of understanding also means researchers cannot judge whether the comparator and the intervention share similar ‘active’ components; if they do, the effects of the intervention may be masked or reduced [9]. In terms of ethics, a trial may be viewed as unethical if the quality and quantity of usual care provided at a trial site falls below that provided elsewhere or below standards specified in clinical guidelines [10].

It is usual care’s potentially heterogeneous nature, and the need to address such methodological and ethical requirements, that have led to some researchers specifying at the start of a trial, what treatment(s) and trial processes will be included in their usual care arm. Researchers have based these comparators on clinical guidelines [11], knowledge of current practice [12, 13], and patients’ views on what would be considered acceptable [14]. Yet, if the treatment chosen differs from what is normally delivered, this could weaken the trial’s external validity [1] and potentially put trial participants at risk [15]. Also, deciding what treatment(s) to include might be a difficult decision, as there may be no clinical guidelines or consensus on which treatments should be considered ‘standard’, and current practices might be less than optimal medical care [16]. In addition, there may be insufficient evidence to establish what usual care is [17], and factors such as the feasibility of standardising care across sites also need to be considered [1, 16]. Thus, both of what we will refer to in this paper as ‘unrestricted’ and ‘defined’ usual care comparators (see the ‘Glossary’ section), have their strengths and weaknesses.

There is literature detailing when a usual care comparator should be used [18, 19], and researchers have published protocols of trials that include usual care arms and justified their content [20, 21]. Currently, however, there is no guidance detailing how researchers should decide the content of usual care comparators when designing trials of complex interventions, and what should inform this decision. The aim of this methodology review is to assess current thinking around what factors should drive this decision and what actions should be taken whilst making it. Its focus and design were informed through a discussion with seven patient and public involvement members prior to submitting the application for funding, and one of these members was a co-applicant on the study, attended team meetings, and is an author on this paper (TY).

## Methods

We registered the protocol with PROSPERO (CRD42022307324). The reporting of this review was guided by the PRISMA guidelines [22].

### Searches and screening

Four electronic bibliographic databases (MEDLINE, Embase, CINAHL and PsycINFO) were searched from the inception of databases to 7th January 2022. A comprehensive search strategy was developed and tested with support from an information specialist (SaD). Searches included both MeSH and free text terms relating to usual care and synonyms, and methodology-related terms, such as methodology and research design. The parent Medline search strategy can be found in Additional file 1: Appendix 1. In addition to searching databases, we also emailed experts in the field and used social media to contact experts, asking them to share relevant literature.

After deduplication, we exported references into Rayyan [23] to screen results from the database searches. Titles and abstracts and the full texts were independently screened by two reviewers (KT and SD) against study criteria. Any disagreements were resolved through discussion and, where necessary, in consultation with a third reviewer (AH). Reference lists of included articles were hand searched, and forward citation searches conducted, to identify additional relevant articles.

### Inclusion and exclusion criteria

We included methodology papers, reviews, book chapters, and articles based on case studies that described how to identify or develop usual care comparators in trials of complex interventions. These trials could be in any population and based in primary, secondary or social care, or in public health. We used the MRC’s definition of complex interventions [24] and therefore excluded papers detailing comparators in trials evaluating medicines (e.g. drugs or vaccines) and which focused only on treatment outcomes and not, for example, improving adherence. No language restrictions were applied if an English language abstract was available for initial screening.

### Quality assessment of included articles

We did not conduct a risk of bias assessment, as our aim was to review methodological literature to understand current thinking around how researchers should identify or define usual care when planning a trial. Thus, it would not have been appropriate to do so.

### Data extraction strategy

A customised data extraction table was developed in Microsoft Word. We extracted data on what principles, considerations and evidence should drive the decision about what usual care comparators should include (we defined these data as ‘decision drivers’), and what steps or tasks researchers should undertake when making this decision (we referred to these as ‘actions’). We also extracted details about the articles and the definitions of usual care used.

The data extraction table was tested on a random sample of two papers and refined. Data were extracted independently by two reviewers (KT and SD), and any discrepancies were resolved through discussion with a third reviewer (AH).

### Data synthesis and presentation

We followed Popay et al.’s guidance on narrative synthesis using the general framework proposed [25]. Specifically, element 2: the development of a preliminary synthesis (developing an initial description of results) and element 3: exploring relationships within and between studies. This framework allowed us to shape both our synthesis and discussion of the included studies.

When synthesising details about the articles, we documented within a table information such as, where the article had been published, what terms had been used to refer to a usual care comparator and how this arm had been defined. When synthesising data on drivers and actions, we aimed to provide a narrative rather than a quantitative overview. This was because the articles included in the review varied in their focus and structure, so it was not possible to compare them directly or list how many articles mentioned a specific driver or action. Also, in many cases, it was our analysis and interpretation of the data, rather than an explicit statement in the article, that resulted in text being viewed as detailing a driver or an action.

As we identified individual drivers, it became apparent some related to the context in which a trial would be implemented, whilst others related to what a trial needed to do to address its aim and remain ethical. We therefore labelled drivers as either ‘context drivers’ or ‘trial drivers’, and as we continued to synthesise the data, began to consider how individual drivers might relate and affect each other. Similarly, we realised actions could be grouped according to when they needed to be undertaken, during the decision-making process, to decide the content of a usual care comparator.

In the “Results” section, we describe the included articles, before detailing drivers and actions.

## Results

### Included articles

**Fig. 1:**
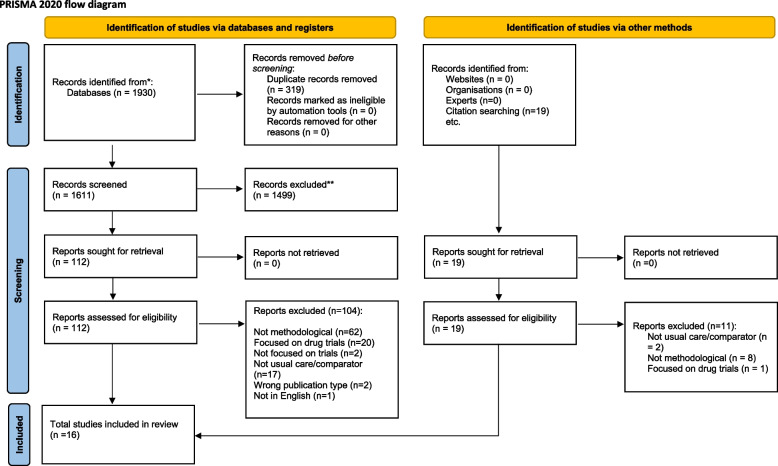
PRISMA 2020 flow diagram

We identified 1930 articles from searching databases. After de-duplication, 1611 titles and abstracts were screened. One hundred twelve articles were included for full-text screening and 16 were included in the review (Fig. 1).

Of the 16 included articles, all but one were published by authors based in the USA [2, 4, 10, 12, 15–17, 26–33]. The exception was published by authors based in Canada [9]. Thirteen focused specifically on the use, design and implications of usual care comparators [2, 4, 9, 10, 15–17, 26, 27, 29, 31–33], and three discussed the selection of control groups more broadly but included text or a specific section on usual care comparators [12, 28, 30].

Six papers described themselves as reviews [9, 10, 15, 17, 27, 31]. Nine papers did not define themselves or simply said ‘this article’. We defined them as ‘discussion/methodological’ articles [2, 4, 12, 16, 26, 28–30, 33], as they discussed, for example, situations when unrestricted usual care may not be ethically acceptable [26], and how the vulnerability of the target population should inform the use and design of usual care controls [4]. The remaining included paper described an empirical study that identified current treatments for adolescent suicide attempters and then discussed the implications of the study findings on the use and design of usual care arms [32]. Whilst this was the only article based on an empirical study, five of the other included papers described individual studies to illustrate points made [4, 12, 16, 28, 29].

In terms of topic area, four articles were published in the area of critical care [9, 15, 29, 31], five in the areas of mental health, i.e. psychotherapy [26], psycho-oncology [17], genetic counselling [27], suicide prevention [4, 32], and one in the area of Type 2 diabetes [33]. The remaining articles focused on trial or intervention types: experimental studies [12], behavioural effectiveness trials [28], clinical trials [16], behavioural interventions [10], non-pharmacologic interventions [2] and psychological interventions [30] (Table 1).

**Table 1:** Details of included articles

Author and date	Subject area	Terms used for usual care	Text defining term(s) used
Angriman et al., 2019 [9]	Critical care	Usual care Protocolized usual care Unrestricted usual care	*Depending on the clinical situation, ****usual care**** may be relatively narrow (e.g., a red blood cell transfusion threshold of 70 g of hemoglobin per liter in general critical care populations) or it may be relatively broad (e.g., the timing of strategies to treat patients with severe hypoxemia, or the amount of fluid given for resuscitation of patients in septic shock). ‘****Protocolized’ usual care**** would select from one of the practice patterns that comprise usual care; ****unrestricted usual care**** would not impose any such limits.* (page 499)
Applefield et al., 2020 [15]	Critical care	Usual care	*Properly designed head-to-head comparisons of contemporary care can improve clinical decision making by better quantifying relative risks and benefits. However, for such research to be informative, at least one arm must be truly representative of current medical practice. Some trials purporting to compare ****usual care**** practices may not accurately reflect those practices.* (page 110)
Arch and Stanton, 2019 [17]	Psycho-oncology	Usual care	*Within psycho-oncology trials, ****usual care**** (UC) represents a common and important control condition. When we need to know whether a new psycho-oncology intervention improves care or cost beyond the offerings already in place, UC represents the most logical control condition.* (page 1592)
Arean and Alvidrez, 2002 [26]	Psychotherapy	Usual care Treatment as usual	*Because effectiveness research is generally concerned with the effectiveness of new interventions compared to existing treatment, the typical comparison condition in this research is ****usual care****, sometimes ****called treatment as usual (TAU)****.* (page 63)
Barkauska et al., 2005 [12]	Experimental studies	Usual care Devised usual care	**Usual care** – *because of ethical concerns, health care providers are reluctant to discontinue a usual treatment unless a new intervention is proven to be more beneficial. In such cases, investigators may need to add the experimental intervention to treatments already being provided to all participants.* (page 354) **Devised usual care**—*a usual treatment, typical of the approaches used in the field and administered in a manner parallel to the experimental intervention.* (page 355)
Biesecker et al., 2020 [27]	Genetic counselling	Usual care	*‘****usual care’**** to refer to the standard care offered in such a control group.* (page 43)
Brigham et al., 2009 [28]	Behavioural interventions for substance abuse	Treatment as usual	*in this paper we discuss ****treatment as usual**** as the standard practice of the community treatment providers.* (page 4)
Dawson et al., 2009 [16]	Clinical trial design	Usual care	*We use the term “****usual care****” to describe the care commonly given by practitioners in a community to avoid any legal or normative implications of the term ‘‘standard of care.’’* (page 1)
Degenholtz et al., 2002 [4]	Suicide Prevention	Treatment as usual	*PROSPECT randomly assigns practices to either an intervention arm (which includes assessment and care plan recommendation by a mental health specialist) or to a ****TAU arm****, in this case consisting of usual medical care with the addition of screening and assessment services.* (page 44)
Freedland et al., 2011 [10]	Trials of Behavioural interventions	Existing practice control conditions Treatment as usual Usual care Enhanced usual care Constrained usual care Standardized treatment regimen Standard of care Uniform or protocol-driven standard of care individualized standard of care Inadequate care	***Existing practice (EP) control conditions**** are used to compare experimental interventions to existing treatments or clinical practices*. (page 3) ***Treatment as usual (TAU) control groups**** are used to compare experimental interventions to treatments that are already used in clinical practice…****Usual care (UC)**** is a roughly equivalent term that is used much more often than TAU in medical trials and in behavioral medicine.* (page 3) ***Enhanced usual care (EUC)**** condition, usual care is systematically improved by the research protocol to overcome ethical or methodological problems that would accompany ordinary UC.* (page 3) ***Constrained usual care (CUC****), in which nonstudy care is restricted in some way.* (page 3) ***Standardized treatment regimen (STR)****, in which the same clinical care or treatment(s) are administered in the same way to all participants. Standardization does not necessarily mean that every patient receives identical treatment. Instead, each patient may be treated according to a standardized protocol or care path.* (page 3) *In ****standard of care (SOC) control groups****, participants receive state-of-the-art, evidence based, guideline-adherent clinical care. SOC is a naturalistic condition when patients are recruited from settings that provide it routinely… SOC may have to be imposed by enhancement of usual care when patients are recruited from less stellar settings.* (page 3) *A ****uniform or protocol-driven standard of care (uSOC)**** produces the best clinical outcomes for some conditions, but an ****individualized standard of care (iSOC)**** is best for others.* (page 3) *The most problematic EP control condition might be called ****inadequate care (IC)****, reflecting the inferior healthcare services to which underserved, uninsured, or captive patient populations may be relegated.* (page 3)
Macklin and Natanson, 2020 [29]	Critical care	Usual care	*“****usual care****”— current treatments clinicians use in caring for patients.* (page 31)
Mohr et al., 2009 [30]	Trials of psychological interventions	Treatment as usual Enhanced treatment as usual	*A ****TAU control**** uses the routine intervention(s) ordinarily provided by clinicians in the settings from which participants are recruited.* (page 279) *The outcomes of TAU may also include variability from sources other than the treatment itself… These unwanted sources of variance can be limited by standardizing them across treatment arms. For example, standardizing the identification of study participants across treatment arms… Such ****‘enhanced’ TAU**** conditions can focus control on treatment effect.* (page 279)
Silverman and Miller, 2004 [31]	Critical care	Standard care Unrestricted standard of care control group Protocolized control groups	*We use the phrase “****standard care****” practices to refer to routine intensive care unit practices … such practices represent critical care treatments that physicians currently provide to their patients, making them the normative baseline to which other proposed strategies should be compared. We use the phrase “****standard of care control group****” to refer to a control group that represents the range of standard practices.* (page 853) *Some critical care RCTs compare an experimental strategy with a control group representing the broad range of standard practices in which the selection of treatment for individual patients is at the discretion of the attending physicians. We call this type of control group an ****unrestricted standard of care control group****.* (page 853) *Due to the variations in standard practices and multiplicity of interventions used in critical care practice, many critical care trials impose constraints on study and nonstudy interventions in both the experimental and control groups. Accordingly, subjects in the control groups are managed according to protocols that specify and restrict the parameters of standard practices… Depending on the extent of variation in standard practices and the nature of the constraints imposed by a protocol on these practices, ****protocolized control groups**** may differ in the extent to which they represent standard of care practices.* (page 854)
Spirito et al., 2002 [32]	Suicide prevention	Treatment as usual	*The comparison groups used in these studies varied, and in fact, two studies used a no-contact control group. Almost half (n* = *8) of the studies randomized comparison group patients to ****treatment-as-usual (TAU)**** in the community, that is, treatments that adhere to some community standard of acceptable practice.* (page 41)
Thompson and Schoenfeld, 2007 [2]	Trials of nonpharmacologic interventions	Usual care	*the terms “best current” therapy or “standard of care” are problematic as they imply a uniform or proven practice standard. We prefer the descriptive term “****usual care****” to describe *de facto* clinical care without any value judgment. (*page 577)
Young et al., 2020 [33]	Type 2 diabetes	Usual care	*the term ****usual care**** (also referred to as routine care, control case, or standard treatment) describes a wide spectrum of care practices. (page 126)*

### Terminology used and definitions of usual care

When first defining usual care, nine articles used the term usual care [2, 9, 12, 15–17, 27, 29, 33], four used the term treatment as usual [4, 28, 30, 32], two used both of these terms [10, 26], and the remaining article used the term standard care [31]. All of these terms were used to refer to existing treatments or health care practices used in practice (Table 1). This suggested that they are used interchangeably within the literature, and certainly text such as ‘the term usual care (also referred to as routine care, control case, or standard treatment)’ ([33], page 126) and ‘usual care, sometimes called treatment as usual’ ([26], page 64) supports this suggestion.

However, Dawson et al. [16] and Thompson and Schoenfeld [2] both included sections on terminology and detailed why they had chosen the term usual care. Dawson et al. explained they had used it ‘to avoid any legal or normative implications of the term “standard of care.”’ (page 1) and Thompson and Schoenfeld wrote ‘the terms “best current” therapy or “standard of care” are problematic as they imply a uniform or proven practice standard. We prefer the descriptive term “usual care” to describe de facto clinical care without any value judgment.’ (page 577).

Some of the articles used specific terms to refer to a defined usual care comparator, such as protocolised usual care [9] and devised usual care [12] (Table 1).

### Drivers and actions informing the content of usual care

**Fig. 2:**
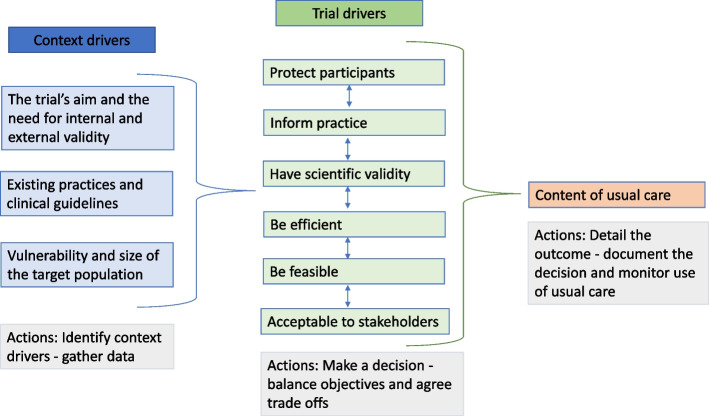
Drivers and actions

Synthesis of the text extracted on drivers indicated that the following should drive decisions about the content of a usual care arm: a trial’s purpose and the need for internal and external validity; existing practices; the existence and content of clinical guidelines; and vulnerability and size of the target population. We viewed these as ‘context drivers’, as they came from the context in which a trial would be implemented. We also identified ‘trial drivers’, which related to the requirements of ethical research that stipulate trials must protect their participants, produce finding that inform practice, have scientific validity and be efficient, feasible and acceptable to stakeholders. These context and trial drivers are detailed below, under individual subheadings, and listed in Fig. 2.

Through synthesising and reflecting on our findings, we visualised that when deciding the content of a usual care arm, context drivers needed to be identified and considered before trial drivers, as they would influence how the trial drivers were prioritised within a trial, which would then inform the content of the usual care comparator. For example, if the target population was viewed as vulnerable, and/or existing care viewed as substandard compared to clinical guidelines (context drivers), ensuring participant safety within a trial (trial driver) would be a high priority and one that might result in a decision to define or enhance usual care. We also realised that when accounting for different trial drivers, tensions might arise between them. For example, the need to protect participants might reduce the extent to which findings would inform current practice, if the former required usual care to be enhanced beyond what was normally provided in real-world settings. Thus, there was a sense of needing to balance or trade trial drivers against one another when determining the content of usual care (this is indicated by the arrows included in Fig. 2, between trial drivers).

Synthesis of text detailing what actions researchers should undertake when deciding what usual care should include, showed these could be categorised as actions to gather data to understand the context in which a trial would be implemented; actions to support the process of deciding which trial drivers should be prioritised within a trial and what trade-offs would be made, and what actions should be taken having decided the content of usual care.

### Context drivers

#### The trial’s aim and the need for internal and external validity

Unrestricted usual care comparators were described as essential to pragmatic effectiveness trials where the aim was to establish the utility of an intervention compared to current practice [26]. However, Brigham et al. [28] highlighted that if the experimental intervention needed to be substantially altered for it to be implemented in a real-world setting, the aim of the trial moved away from assessing its effectiveness towards assessing its efficacy. This in turn raised the importance of the trial’s internal validity, potentially requiring some control over what the intervention was compared against, and therefore what was delivered as a comparator.

The need to balance internal and external validity within a trial was also discussed in relation to the implications of a trial resulting in a type I or type II error, in terms of whether the decision to implement or further evaluate an ineffective treatment (type I error) would have greater negative impact on stakeholders (e.g. patients and providers) than concluding a treatment was not effective when in fact it was (type II error) [30].

The specific focus of a trial, in terms of whether it assessed the use of services or the effectiveness of a treatment compared to another, was also noted as important. If the former, unrestricted usual care might be appropriate but if the latter, the usual care arm might need to include processes to ensure participants in the comparator arm accessed existing practices [26].

#### Existing practices and clinical guidelines

Several articles mentioned existing practices should inform decisions about comparator content. If existing care was variable within or across trial sites, or between providers, in terms of quality, availability and/or accessibility to participants, then usual care should be standardised or enhanced within a trial [12, 26]. The same applied if existing practices were substandard to some local or national standard [26]. If current practice at a site was no treatment, no treatment would be an acceptable comparator if no guidelines exists but would be unacceptable if guidelines existed showing that practices elsewhere were more effective than no treatment [26]. A no treatment usual care arm was also unacceptable if care was already being provided, as in such circumstances it would be unethical to withhold care from trial participants [10, 12].

If there was so much variation in current practice that ‘‘standard’ care appears to be a misnomer’ ([32], page 46), and therefore viewed as an inadequate control group against which to evaluate another intervention, then usual care should be defined. Similarly, if usual care was viewed as too weak, too atypical, too variable in content, and too different from the intervention to act as an adequate comparator, it should be defined [12]. The effectiveness of existing care was also highlighted. For example, Degneholtz et al. [4] discussed a trial in suicide prevention where existing practices had been linked to high suicide rates due to unrecognised, untreated or undertreated depression, and the experimental intervention aimed to improve access to care considered beneficial. The vulnerability of the target population, alongside the possibility that clinical equipoise might not exist between trial arms, meant usual care in this trial was enhanced to meet the demands of participant protection and scientific rigour.

#### Vulnerability and size of the target population

Already noted is the article by Degenholtz et al. [4] where vulnerability of the target population, alongside inadequate usual care practices, resulted in usual care being enhanced within a trial. In addition to this article, vulnerability of the target population influencing the content of usual care was also highlighted by three of the four articles published in the area of critical care [15, 29, 31]. These articles argued that creating a comparator that did not reflect current practice, and/or restricted the extent to which care could be tailored to the specific needs of an individual or subgroup, could result in trial participants receiving inappropriate or suboptimal care. Two of these articles provided evidence to support their arguments, by detailing three critical care trials where mischaracterising usual care had resulted in significant or fatal consequences for participants in the usual care arm [15, 29].

In terms of the size of the target population informing the content of usual care, the availability of potential trial participants could be a driver if there was a limited number of potential trial participants with the disorder or condition of interest, as conducting a large trial might not be feasible. In this situation, where the size of a trial could be limited, a researcher may want to define usual care to restrict its content and heterogeneity in order to reduce sources of variation within the trial. Reducing sources of variation would strengthen a trial’s ability to detect, if present, differences in outcomes between trial arms [31]. Defining usual care would also ensure that practices included in the comparator did not overlap with those delivered as part of the intervention. This would also strengthen the trial’s ability to detect differences.

### Trial drivers

#### The need to protect participants, inform practice, have scientific validity and be efficient

Silverman et al. [31] argued that the content of a usual care arm should be driven by the need to conduct ethical research. They commented that to be ethical, a trial must protect participants, be of clinical value (i.e. generate knowledge that has the potential to enhance practice), have scientific validity (i.e. have sufficient methodological rigour to generate valid results) and be efficient (i.e. avoid wasting resources). As these objectives may conflict, the decision about choice of comparator might involve trade-offs and compromises. For example, as Silverman et al. [31] explained, defining usual care might increase a trial’s scientific validity, as it could reduce variation in usual care, ensuring the comparator does not overlap or drift towards the experimental intervention, maximising a trial’s ability to detect differences in outcomes between trial arms. It might also be more efficient if the defined comparator meant a smaller sample size was needed, as it would reduce time and costs required. However, it might be of less clinical value and pose greater risks to participants if it does not accurately represent existing practices.

The requirements of ethical research informing the content of a usual care comparator were also highlighted by Degenholtz et al. [4]. They described a trial where entire populations were medically screened to identify individuals for study inclusion, and thus resulted in information about an individual’s health that otherwise would not have been known. This led to researchers having an ethical responsibility to monitor participants in the control arm to ensure that any adverse consequences of their trial participation or medical condition, were addressed.

#### The need to be feasible and acceptable to stakeholders

The content of a usual care arm could be informed by the finances available and the size of the target population, as both factors would affect how feasible it would be to conduct a large trial. For example, if finances were limited and/or there was a limited number of potential trial participants, a trial might be restricted in terms of its size and may need to define usual care in order to have sufficient power to identify differences between trial arms [31].

Whether a trial was feasible would also depend upon stakeholder engagement and successful recruitment of trial participants. Its design, therefore, must be acceptable to those implementing the comparator arm and to potential trial participants. Clinicians or providers might object to usual care being defined if they think tailoring treatment to the needs of specific individuals would result in better outcomes [16], and the target population might have a preference of unrestricted usual care [9].

When considering the feasibility of defining usual care, researchers might also want to consider where variability in current practices stems from. If it stems from various factors, e.g. age and needs of the patient, resources availability and/or clinician preferences, defining usual care could be particularly challenging [33].

### Actions to undertake when making a decision

Some articles explicitly or implicitly mentioned actions that researchers could undertake to ensure their usual care comparator would be appropriate for their specific trial. These actions gathered data on the context drivers detailed above, facilitated the decision-making process that would then occur to determine priorities within a specific trial, and detailed what should be done once a decision had been made about the content of usual care (Table 2).

**Table 2:** Actions to inform and document the content of usual care

*Identify context drivers — gather data*
Characterise and document existing practices
• Understand, appraise and document existing practices
• Include institutions participating in the study in the characterisation of current practices
• Establish whether potential trial participants can access existing practices and how they currently manage the target problem
Know current clinical guidance
• Identify and read relevant best-practice guidelines
*Make a decision — balance objectives and agree trade offs*
Assess existing practices
• Consider the extent to which existing practices are evidence-based and vary between trial sites
• Consider whether the usual care arm will contribute to meaningful inferences about the experimental intervention
• Consider the extent to which existing practices overlap with the experimental intervention
• Compare existing practices to the experimental intervention in terms of intensity and duration
• Consider the extent to which existing practices at each trial site reflect national or community standards
• Acknowledge disagreements about what usual care involves and identify source of disagreement
Consider alternative comparators
• Consider advantages and limitations of usual care controls compared to alternative comparators
• Develop criteria to review possible comparators
• Discuss alternative comparators with policymakers and providers to establish which would be most meaningful and acceptable to them
Think context and the needs of the trial
• Question whether a minimum level of treatment is needed, according to clinical guidelines
• Think systematically about the background conditions in the practising medical community and goals of the trial
• Consider the need for internal and external validity
• Acknowledge practical limitations, e.g. infrastructure, costs, time
*Detail the outcome — document the decision and monitor use of usual care*
Detail the decision-making process
• Document the information and the decision-making process used to decide the content of usual care
Monitor usual care
• Develop methods to monitor usual care and track participants’ use of usual care in the trial

Interestingly, some articles also mentioned actions that related to the design of the trial, rather than informing the content of usual care. These aimed to account for or minimise variation within usual care, without having to define it. For example, randomising providers to deliver usual care or the experimental intervention, to ensure pre-existing skill level was not a factor in any treatment effect [28], and selecting trial sites known to provide care viewed as representative of usual care [26].

## Discussion

The content of usual care comparators in trials evaluating complex interventions should be informed by the aim of the trial and the extent to which internal and external validity is needed; what care is currently being provided in practice, in terms of its content, effectiveness and accessibility to the target population; the existence and content of clinical guidelines; and the vulnerability and size of the target population. In addition to these context drivers, its content will also be driven by the trial’s requirement to protect participants, inform practice, be methodologically robust, efficient, feasible, and be acceptable to stakeholders. All of these latter trial drivers need to be met for a trial to address its aim, whilst remaining ethical and feasible. This may be difficult to achieve, as one driver might indicate the need to define usual care, whilst another might suggest it should be unrestricted. Some of the actions we identified could help researchers balance such tensions, for example, developing criterion to review possible comparators, and discussing alternative comparators with policymakers and providers. However, as others have noted, trade-offs may need to be made and the most appropriate comparator will be the one that best fits with the purpose of that specific trial [10].

To date, much of the discussion around the need to clarify the content of usual care has stemmed from the recognition that its content might vary between trial sites and that its content will affect the effect size found, so needs to be carefully described when interpreting and reporting trial findings [6, 30, 34]. Our review highlights reasons for it being defined from the start of a trial and supports the observation that the ethical requirements of research can mean usual care needs to be altered [35]. In addition, whilst we noted variation in the content and accessibility of usual care as a key driver, we also noted that if existing practices were viewed as ineffective, or below standards set locally or via clinical guidelines, then usual care might need to be defined. We also noted that usual care might be enhanced to ensure clinical equipoise between trial arms, or because recruitment processes in a trial resulted in information about an individual’s wellbeing, which otherwise would not have been known. Thus, it is not simply about variability in usual care but also what is viewed as appropriate care when compared to external standards and to the experimental intervention, and how trial processes might affect the population from which it is recruiting.

Whilst the focus here was on how to determine the content of usual care comparators, others have emphasised that study processes also need to be considered when designing trial arms. For example, it has been argued that differences can exist between control and intervention arms relating to the ways in which individuals’ access and engage with care provided within them. As these differences could affect treatment outcomes, researchers should aim to reduce them when designing trial arms [36].

Considering how important the content of a usual care arm is to the ethical and methodological conduct of a trial, it is interesting that such little attention has been given to how it should be decided. This could be because, as Burns [37] argues, our understanding of trials has been limited by the view that the experimental arm represents ‘the’ intervention, and the usual care control simply a necessary structure to facilitate the trial. Burns suggests that future trials are developed, interpreted and reported as a comparison of two interventions, and that the term ‘treatment as usual’ should not be used as it implies there is some consistent background practice again which any new intervention can be evaluated. We agree with both of these statements and hope our review goes some way towards helping researcher actively consider and question the content of usual care comparators and move towards viewing them as complex interventions in their own right.

In terms of terminology, we noted that some researchers have considered what terminology they use when referring to a usual care comparator, and that specific terms have been used when referring to a defined usual care arm. Like others though, we noted researchers use a range of terms to describe usual care and interpret the usual care concept differently [35]. Perhaps if the content of usual care arms is given more thought, researchers will start to describe the care provided within them. Detailing what care is provided would enable researchers to meet requirements of reporting statements, such as CONSORT [38] and TIDieR [39], which ask researchers to clearly detail both intervention and comparator arms, and which a recent review of published trial protocols shows are not being met, with researchers providing limited or no information on the content of their usual care arms [40].

A potential limitation of our review is that we may not have identified all relevant articles because the term ‘usual care’ has not been consistently used. That said, our search strategy included terms such as standard care, usual care, usual medical care, and comparison group to address this. Differences between articles in terms of their structure, focus and content meant we adopted a narrative summary approach when synthesising and presenting findings on drivers and actions. This enabled us to provide an overview of current thinking about how to select or develop a usual care arm, but we are aware that we extracted and categorised data as drivers and actions, and these were not terms used within the articles themselves. We are also aware this methodology review gives no insight into the reality of designing trial comparator arms and that some of the actions detailed could be very challenging to address. For example, as usual care can differ between patients and practitioners, across clinical sites and over time, characterising and assessing existing care practices could be very difficult.

If the long-term ambition is to develop guidance to support researchers designing trials of complex health interventions, then an understanding of this reality and assessing the extent to which such guidance could be applied in practice would be needed to ensure it is relevant and applicable. Future research could entail conducting in-depth interviews with trialists and wider stakeholders (e.g. Directors of funding committees and trial units, Research Design Service staff, and health care practitioners) to explore their views and, where appropriate, experiences of defining usual care. Such work could highlight drivers and actions not yet detailed within the existing literature, which is limited, and indicate the feasibility of undertaking some of the actions discussed, and the most efficient or effective way of addressing them. Interviews with researchers who have defined usual care could also provide insights into the impact of defining usual care on a trial’s implementation and relevance to real-world practice. Once such in-depth work had been completed, a consensus exercise could be undertaken with researchers and wider stakeholders to agree which drivers and actions should be detailed in the guidance, and what information should be provided about how best to undertake the decisions and actions faced when defining usual care. Prior to finalising this guidance, it could be applied to two or three case study trials to assess feasibility of implementation and its acceptability to trialists.

## Conclusions

Both the context within which a trial is conducted, and the needs of that trial, will determine what its usual care comparator should include. Compromises might need to be made, as tensions may arise when accounting for different drivers. What is important is that researchers actively think about the content of their usual care arm, acknowledge its strengths and limitations, and justify its selection. To be able to do this, primary research is needed at the design stage of a trial to understand what care is currently being provided in practice, what the needs of the target population are, and what clinical guidelines exist. In addition, the decision-making process that will then occur to determine content of usual care, will require engagement with stakeholders, and members of the research team to consider the needs and priorities of their trial, and what structural, population and financial factors might constrain what is possible. Such work requires time and resources and could be done as part of a feasibility study.

### Supplementary Information

**Additional file 1: Appendix 1.** Parent search strategy.

## Data Availability

The dataset used and analysed during the current study are available from the corresponding author.

## Glossary

Complex health interventions: Interventions that are complex due to their properties, such as the number of components involved, the range of behaviours targeted, the expertise and skills required to deliver/receive it, number of groups/settings/levels targeted, or the permitted level of flexibility of the intervention or its components
Defined usual care comparator: A comparator that includes only treatments and care processes the researchers have selected to be included in this trial arm
Explanatory trials: Trials undertaken in an idealised setting that aim to assess the efficacy of an intervention to demonstrate its beneficial effect
External validity: The extent to which trial findings can be generalised to other situations, people, settings, and measures
Internal validity: The extent to which the trial can establish a trustworthy cause-and-effect relationship
Pragmatic trials: Trials undertaken in real-world settings that aim to assess the effectiveness of an intervention to support a decision about whether to deliver it in practice
Type I error: Concluding that an intervention is effective, when it is not
Type II error: Concluding that an intervention is not effective, when it is
Unrestricted usual care comparator: A comparator that includes the full range of treatments and care processes available in practice

## References

[1] Zuidgeest MGP, Welsing PMJ, van Theil GJMW, Ciaglia A, Alfonso-Cristancho R, Eckert L (2017). Pragmatic trials and real world evidence: paper 5. Usual care and real life comparators. J Clin Epidemiol..

[2] Thompson BT, Schoenfeld D (2007). Usual Care as the Control Group in Clinical Trials of Nonpharmacologic Interventions. Proc Am Thorac Soc.

[3] Ayling K, Brierley S, Johson B, Heller S, Eiser C (2015). How standard is standard care?. Psychol health.

[4] Degenholtz HB, Parker LS, Reynolds CF (2002). Trial design and informed consent for a clinic-based study with a treatment as usual control arm. Ethics Behav.

[5] Staines GL, McKendrick K, Perlis T, Sacks S, De Leon G (1999). Sequential assignment and treatment-as-usual. Alternatives to standard experimental designs in field studies of treatment efficacy. Eval Rev..

[6] de Bruin M, Viechtbauer W, Hospers HJ, Schaalma HP, Kok G (2009). Standard care quality determines treatment outcomes in control groups of HAART-adherence intervention studies: Implications for the interpretation and comparison of intervention effects. Health Psychol.

[7] Mant D (2008). The problem with usual care. BJGP.

[8] Somerville S, Hay E, Lewis M, Barber J, Van der Windt D, Hill J, Sowden G (2008). Content and outcome of usual primary care for back pain: a systematic review. BJGP.

[9] Angriman F, Masse MH, Adhikan NKJ (2019). Defining standard of practice: pros and cons of the usual care arm. Curr Opin Crit Care.

[10] Freedland KE, Mohr DC, Davidson KW, Schwartz JE (2011). Usual and unusual care: existing practice control groups in randomised controlled trials of behavioural interventions. Psychosom Med.

[11] Bosmans J, de Bruijne M, Van Hout H, Van Marwijk H, Beekman A (2006). Bouter L et al Cost-Effectiveness of a Disease Management Program for Major Depression in Elderly Primary Care Patients. J Gen Intern Med.

[12] Barkauska VH, Lusk SL, Eakin BL (2005). Selecting Control Interventions for Clinical Outcome Studies. West J Nurs Res.

[13] Mazya AL, Eckerblad J, Jaarsma T, Hellstrom I, Krevers B, Milberg A (2013). The ambulatory geriatric assessment- a Frailty Intervention Trial (Age-FIT)- A randomised controlled trial aimed to prevent hospital readmissions and functional deterioration in high risk older adults: a study protocol. Eur Geriatr Med.

[14] Brooks S, Peters T, Campbell R, Featherstone K, Neal D, Abrams P, Donovan J (2003). Including a ‘no active intervention’ arm in surgical trials is possible. J Health Serv Res Policy.

[15] Applefield WN, Wang J, Klein HG, Danner RL, Eichacker PQ, Natason C (2020). Comparative effectiveness research in critically ill patients: risks associated with mischaracterising usual care. Criti Care and Resusc.

[16] Dawson L, Zarin DA, Emanuel EJ, Friedman LM, Chaudhari B, Goodman SN (2009). Considering usual medical care in clinical trial design. PLoS Med.

[17] Arch JJ, Stanton AL (2019). Examining the “usual” in usual care: a critical review and recommendations for usual care conditions in psycho-oncology. Support Care Cancer.

[18] Freedland KE (2020). Purpose-guided trial design in health-related behavioral intervention research. Health Psychol.

[19] EUnetHTA guideline: comparators and comparisons. EUnetHTA-21-D4.3-Comparators-and-comparisons-Project-Plan-v1.0.pdf. Accessed 5 Oct 2023.

[20] Egede LE, Davidson TM, Knapp RG, Walker RJ, Williams JS, Dismuke CE, Dawson AZ (2021). HOME DM-BAT: home-based diabetes modified behavioral activation treatment for low-income seniors with type 2 diabetes—study protocol for a randomized controlled trial. Trials.

[21] Richards S, Dickens C, Anderson R, Richards DA, Taylor R, Ukoumunne O, Kessler D, Turner K, Kuyken W, Gandhi M, Knight L, Gibson A, Davey A, Warren F, Winder R, Wright C, Campbell J (2016). Assessing the effectiveness of enhanced psychological care for patients with depressive symptoms attending cardiac rehabilitation compared with treatment as usual (CADENCE): A study protocol for a pilot cluster randomised controlled trial and qualitative interview study. Trials.

[22] Page M, McKenzie JE, Bossuyt PM, Bortron I, Hoffman T, Mulrow CD (2021). The PRISMA 2020 statement: an updated guideline for reporting systematic reviews. BMJ.

[23] Ouzzani M, Hammady H, Fedorowicz Z, Elmagarmid A (2016). Rayyan-a web and mobile app for systematic reviews. Syst Rev.

[24] Skivington K, Matthews L, Simpson SA, Craig P, Baird J, Blazeby J (2021). A new framework for developing and evaluating complex interventions: update of Medical Research Council guidance. BMJ.

[25] Popay J, Roberts H, Sowden A, Petticrew M, Arai L, Rodgers M (2006). Guidance on the conduct of narrative synthesis in systematic reviews: a product from the ESRC Methods Programme.

[26] Arean PA, Alvidre J (2002). Ethical Considerations in Psychotherapy Effectiveness Research: Choosing the Comparison Group. Ethics Behav.

[27] Biesecker BB, Lillie SE, Amendola LM, Donohue KE, East KM, Foreman AKM (2020). A review and definition of ‘usual care’ in genetic counseling trials to standardize use in research. J Genet Couns.

[28] Brigham GS, Feaster DJ, Wakim PG, Dempsey CL (2009). Choosing a Control Group in Effectiveness Trials of Behavioral Drug Abuse Treatments. J Subst Abuse Treat.

[29] Macklin R, Natanson C (2020). Misrepresenting, “Usual Care” in Research: An Ethical and Scientific Error. The Am J Bioeth.

[30] Mohr DC, Spring B, Freedland KE, Beckner V, Arean P, Hollon SD (2009). The Selection and Design of Control Conditions for Randomized Controlled Trials of Psychological Interventions. Psychother Psychosom.

[31] Silverman HJ, Miller FG (2004). Control group selection in critical care randomized controlled trials evaluating interventional strategies: An ethical assessment. Crit Care Med.

[32] Spirito A, Stanton C, Donaldson D, Boergers J (2002). Treatment-as-Usual for Adolescent Suicide Attempters: Implications for the Choice of Comparison Groups in Psychotherapy Research. J Clin Child Adolesc Psychol.

[33] Young HM, Miyamoto S, Tang-Feldman Y, Dharmar M, Balsbaugh T, Greenwood D (2020). Defining Usual Care in Clinical Trials. Res Gerontoll Nurs.

[34] Warshaw MG, Carey VJ, McFarland EJ, Dawson L, Abrams E, Melvin A (2017). The interaction between equipoise and logistics in clinical trials. Clin Trials.

[35] Smelt AFH, van der Weele GM, Blom JW, Gussekloo J, Assendelft WJJ (2010). How usual is usual care in pragmatic intervention studies in primary care? An overview of recent trials. BJGP.

[36] Turner KM, Percival J, Kessler D, Donovan J (2017). Exploring patients’ treatment journeys following randomisation in mental health trials to improve future trial conduct: a synthesis of multiple qualitative data sets. Trials.

[37] Burns T (2009). End of the road for treatment-as-usual studies?. BJPsych.

[38] Schulz KF, Altman DG, Moher D (2010). CONSORT 2010 Statement: updated guidelines for reporting parallel group randomised trials. BMJ.

[39] Hoffman TC, Glasziou PP, Bouton I, Milne R, Perera R, Moher D (2014). Better reporting of interventions template for intervention description and replication (TIDieR) checklist and guide. BMJ.

[40] Dawson S, Turner K, Huntley A. How do researchers select usual care comparators when designing primary care trials of complex interventions. SAPC conference, July 2023. https://sapc.ac.uk/conference/2023/abstract/how-do-researchers-select-usual-care-comparators-when-designing-primary. Accessed 18 Oct 23.

