# Supplementary material for: Defining usual care comparators when designing pragmatic trials of complex health interventions: a methodology review
Source: Trials. 2024 Feb 12;25:117. doi: 10.1186/s13063-024-07956-7 (PMC10860249; doi:10.1186/s13063-024-07956-7)
Supplement: Supplementary file 1 — Additional file 1: Appendix 1. Parent search strategy. [file 13063_2024_7956_MOESM1_ESM.pdf]

Database(s): **APA PsycINFO**, **Embase** 1974 to 2022 January 07**, Ovid MEDLINE(R) ALL** 1946 to January 07, 2022
Search Strategy:

| # | Searches | Results |
|---|---|---|
| 1 | ((standard care or (usual adj3 care)) and (trials or studies or interventions or trial design or study design or evidence based)).ti. | 279 |
| 2 | ((standard care or (usual adj3 care)) and (choice or choosing or defin* or describ* or descrip* or design* or select* or optimal)).ti. | 139 |
| 3 | ((comparator? or comparison group?) and (choice or choosing or defin* or describ* or descrip* or design* or select* or optimal) and (trials or studies or interventions or trial design or study design or evidence based)).ti,hw,kw,kf,id,ox. | 160 |
| 4 | or/1-3 | 559 |
| 5 | ((standard care or usual care or usual medical care) and (trials or studies or interventions or trial design or study design or evidence based)).ti,hw,kw,kf,id,ox. | 1106 |
| 6 | ((comparator? or comparison group?) adj (choice or choosing or defin* or describ* or descrip* or design* or select* or optimal)).tw. | 896 |
| 7 | 5 or 6 | 2000 |
| 8 | methodology/ | 1628044 |
| 9 | (methodology or research design?).ti,hw,kw,kf,id,ox. | 1859379 |
| 10 | (evidence based medicine and (ethics or quality)).ti,hw,kw,kf,id,ox. | 32637 |
| 11 | evidence based medicine/ and (as topic.hw. or methods.fs.) | 54252 |
| 12 | control groups/ and (as topic.hw. or methods.fs.) | 2957 |
| 13 | experiment controls/ | 0 |
| 14 | or/8-13 | 1933975 |
| 15 | 7 and 14 | 189 |
| 16 | 4 or 15 | 689 |
| 17 | remove duplicates from 16 | 463 |

**Fields searched:** [fs:floating subheading; hw:subject-heading word; id:key concepts (PsycINFO); kw;keyword heading (phrase indexed); kf:keyword heading word; ox:other index terms word (Embase); ti:title; tw:text word (MEDLINE)]
